# Differential inhibition of human cytomegalovirus (HCMV) by toll-like receptor ligands mediated by interferon-beta in human foreskin fibroblasts and cervical tissue

**DOI:** 10.1186/1743-422X-4-133

**Published:** 2007-12-05

**Authors:** Sailesh C Harwani, Nell S Lurain, M Reza Zariffard, Gregory T Spear

**Affiliations:** 1 Department of Immunology/Microbiology, Rush University, Chicago, USA

## Abstract

Human cytomegalovirus (HCMV) can be acquired sexually and is shed from the genital tract. Cross-sectional studies in women show that changes in genital tract microbial flora affect HCMV infection and/or shedding. Since genital microbial flora may affect HCMV infection or replication by stimulating cells through Toll-like receptors (TLR), we assessed the effects of defined TLR-ligands on HCMV replication in foreskin fibroblasts and ectocervical tissue. Poly I:C (a TLR3-ligand) and lipopolysaccharide (LPS, a TLR4-ligand) inhibited HCMV and induced secretion of IL-8 and Interferon-beta (IFNβ) in both foreskin fibroblasts and ectocervical tissue. The anti-HCMV effect was reversed by antibody to IFNβ. CpG (TLR9 ligand) and lipoteichoic acid (LTA, TLR2 ligand) also inhibited HCMV infection in ectocervical tissue and this anti-HCMV effect was also reversed by anti-IFNβ antibody. In contrast, LTA and CpG did not inhibit HCMV infection in foreskin fibroblasts. This study shows that TLR ligands induce an HCMV-antiviral effect that is mediated by IFNβ suggesting that changes in genital tract flora may affect HCMV infection or shedding by stimulating TLR. This study also contrasts the utility of two models that can be used for assessing the interaction of microbial flora with HCMV in the genital tract. Clear differences in the response to different TLR ligands suggests the explant model more closely reflects in vivo responses to genital infections.

## Background

The seroprevalence of human cytomegalovirus (HCMV) in the United States general population is approximately 60% and is even higher in certain socioeconomic groups [1]. HCMV causes severe disease when immunity is suppressed such as in organ transplant recipients or during the later stages of HIV-1 infection [2]. HCMV infection can be transmitted by bodily fluids of infected individuals, including saliva, blood, semen and cervical/vaginal secretions [3]. Infection of infants can occur from exposure to genital fluids during birth and this route of infection can lead to mild to severe neurological sequelae.

Several studies suggest that alterations of genital tract flora in women can affect either initial infection by HCMV or virus replication/shedding. For example, HCMV DNA was detected more frequently in vaginal washings from women with bacterial vaginosis (BV) than in women with normal genital tract flora [4]. BV is an alteration of the female genital tract flora consisting of an increase in both gram negative and gram positive bacteria [5]. Increased HCMV shedding is also associated with concurrent *Chlamydia trachomatis *or *Neisseria gonorrhoeae *infection [6]. Further, infection with *Trichomonas vaginalis*, *N. gonorrhoeae*, and BV are associated with increased intrauterine transmission of HCMV [7]. The cause of the relationship between HCMV, BV and other sexually transmitted infections (STI) is not currently understood although inflammatory changes caused by STI could influence HCMV infection. Inflammation in genital tract infections is in many cases caused by the activation of genital tract cells through Toll-like receptor (TLR)-ligands derived from the pathogens; *N. gonorrhoeae*, *T. vaginalis *and BV flora all have been found to express products that activate TLR [8-10].

In contrast to the studies that show enhancement of HCMV infection or shedding by genital tract infections, other studies show that stimulation through TLR can induce an antiviral state in cells or in animals [11]. For example, replication of HSV-2 in vaginally-infected mice was prevented by intra-vaginal application of purified TLR ligands [12,13]. Similarly, intravenous injection of ligands for TLR3, -4, -5, -7, and -9 inhibit virus replication in Hepatitis B-transgenic mice [14]. The anti-viral effect in these studies was mediated by induction of type I interferons via TLR stimulation [14,15].

In this study, we determined the effect of defined TLR ligands on HCMV replication as a model to better understand how changes in genital tract flora may enhance or inhibit HCMV replication in vivo. Since there are currently no animal models that are susceptible to HCMV, and only certain human cells are susceptible to HCMV infection, the effect of TLR ligands on replication of HCMV was assessed in foreskin fibroblasts (HFF), a previously described in vitro model of HCMV infection [16,17]. The TLR ligand effects were also studied in ectocervical tissue explants since HCMV was recently shown to replicate in this tissue and this may represent a model that more accurately represents in vivo infection by the virus [18].

## Materials and methods

### Cells, tissues, & reagents

Human Foreskin Fibroblasts (HFF) were maintained in culture medium comprised of Minimum Essential Medium (Gibco, Carlsbad, CA) with 10 mM HEPES, 2 mM L-glutamine, 50 μg/ml gentamycin, 2.5 μg/ml amphotericin B, and 10% fetal bovine serum (FBS; BioWhittaker, Walkersville, MD). Cervical tissue was obtained at Northwestern University Medical Center from women undergoing planned hysterectomy for benign disease who had no history of cervical dysplasia. Patient consent was obtained by the treating physicians.

An HCMV clinical strain was engineered to express the Renilla green fluorescent protein under the control of the HCMV major immediate early promoter [19]. The recombinant strain, HCMVPT30-gfp, produces extracellular virus and has similar growth kinetics as the parental strain [18].

Purified lipoteichoic acid (LTA) from *S. aureus *and lipopolysaccharide (LPS) from *E. coli *O11:B4 were obtained from Sigma Aldrich (St. Louis, MO). Poly I:C (PIC) was obtained from Amersham (Piscataway, NJ). CpG 2395, a type C oligodeoxynucleotide, was generously contributed by Coley Pharmaceuticals (Wellesley, MA).

### Treatment and infection of HFF

HFF were grown to 95% confluency in 24-well culture plates and treated with either medium alone or TLR ligands. After 24 h, medium was removed and assayed for cytokines. Cells were washed and CMVPT30-gfp was added (moi = 0.05). Cells were cultured for four hours, the virus inoculum was removed, and cells were cultured an additional 10 days. Monolayers were inspected by epifluorescent microscopy and the number of GFP-positive cells or clusters of cells (foci) was determined.

### Cytokine ELISA

IL-8, IL-10, IL-12, and TNF-α were quantitated in cell culture fluids using CytoSet ELISA kits from Biosource (Carlsbad, California). IFN-α was tested using the IFN-α Module Set from Bender Medsystems (Burlingame, CA). IFN-β was assayed by coating 96-well flat bottom plates (NUNC, Rochester, NY) with 3 μg/ml monoclonal mouse anti-human IFN-β(Chemicon, Temecula, CA). Wells were blocked with 1% bovine serum albumin in phosphate buffered saline for 2 h at 25°C, washed three times and samples added and incubated for 1 h at 25°C. After washing, 3.5 μg/ml polyclonal rabbit anti-human IFNβ (Chemicon) was incubated in wells for one hour at 25°C followed by a 1/10,000 dilution of mouse anti-rabbit coupled to horseradish peroxidase (Chemicon) for 1 h at 25°C.

### IFN-β neutralization

HFF were grown to 95% confluency in 24-well culture plates and treated with either medium alone, Poly I:C (10 μg/ml) or LPS (10 μg/ml) for 24 h. Cells were washed twice with medium and incubated at 37°C in 1 ml of fresh medium for 1 h to maximize removal of residual stimuli. Medium was replaced with 1 ml of complete medium and cells were cultured for an additional 24 hour period. Conditioned supernatants were collected and incubated with either complete medium, rabbit polyclonal anti-IFN-β neutralizing antiserum (Chemicon) (final concentration of 2 × 10^4 ^neutralization units/ml), or normal rabbit serum (NRS) (diluted 1:500 to give the same concentration of rabbit antibody) for 1 h at 37°C. The treated supernatants were then transferred to fresh 24-well culture plates containing confluent naïve HFF, and cultured for 24 hours. Conditioned medium was then removed and HFF challenged with CMVPT30-gfp. Fluorescent cells were counted by microscopy on day 10 after infection.

### Treatment and infection of ectocervical tissue

Cervical tissues were washed extensively, cut into pieces of approximately 3 mm^3^, and cultured in 48 well plates similar to a previously described method [20] except that three ectocervical tissue pieces were cultured in each well of 48 well plates [18]. Tissues were cultured in 0.5 ml medium containing Dulbeco's Modified Essential Medium, 24% Ham's nutrient mixture, 5 μg/ml insulin, 50 μg/ml gentamicin, 100 U penicillin/100 μg/ml streptomycin, 20 mM HEPES, 2 mM L-glutamine, 1 mm sodium pyruvate, and 10% FBS. TLR ligands were added to wells and cultured for 24 hours. Culture supernatants were removed and assayed for cytokines. Tissue pieces were washed and infected with HCMV (10^5 ^pfu per well) for four h at 37°C. Tissue pieces were washed again and then cultured for 10 days.

### PCR quantitation of HCMV infection

Ectocervical explant tissue samples that were infected with HCMV were harvested and weighed. DNA was extracted using the Qiamp DNA Mini kit (Qiagen, Valencia, CA) and assayed by real-time PCR using primers for the DNA Polymerase gene of HCMV [18]. The forward primer used was 5'-CTCGTGCGTGTGCTACGAGA-3' and the reverse primer used was 5'-GCCGATCGTRAAGAGATGAAGAC-3'. A FAM-AGTGCAGCCCCGRCCATCGTTC-TAMRA probe was used for detection of amplified product and a standard curve was generated using known copy numbers of genomic DNA from HCMV strain AD169 (Advanced Biotechnologies Inc., Columbia, MD). Results were expressed as HCMV copies/mg tissue.

### Expression of TLR by HFF and ectocervical explant tissue

HFF, tissue, HEK293 were lysed and RNA extracted using the RNeasy Mini Kit (Qiagen, Stanford Valencia, CA). cDNA was made from 1 μg RNA from each cell type using the RT-PCR Kit from Clontech (Palo Alto, CA). The TLR primers were designed using Clone Manager Primer Software (4 Sci-ed, Durham, NC) based on gene sequences obtained from GeneBank (National Center of Biotechnology Information, NIH, Bethesda, MD). The primers were; TLR2 (F 5-CTCCAATCAGGCTTCTCT-3, R 5-TCAGTATCTCGCAGTTCC-3); TLR3 (F 5-GCATTCGGAATCTGTCTCTG-3, R 5-ATTCCTGGCCTGTGAGTTCT-3); TLR4 (F 5-GATGCCAGGATGATGTCT-3, R 5-CCGCAAGTCTGTGCAATA-3); TLR9 (F 5-TACCTTGCCTGCCTTCCTAC-3, R 5-CAACACCAGGCCTTCAAGAC-3); and GAPDH (F 5-GAAGGTGAAGGTCGGAGTC-3, R 5-GAAGATGGTGATGGGATTTC-3). Amplification was carried out using a GeneAMP Thermocycler (Perkin Elmer, Norwalk, CT) with a thermocycler profile as follows; Stage 1, 94°C (5 min); stage 2, 35 cycles of 94°C (45 sec), 62°C (45 sec) and 72°C (1 min) and Stage 3, 72°C (10 min).

## Results

### TLR3 and TLR4 ligands but not TLR2 or TLR9 ligands induce IL-8 secretion in foreskin fibroblasts

**Figure 1:**
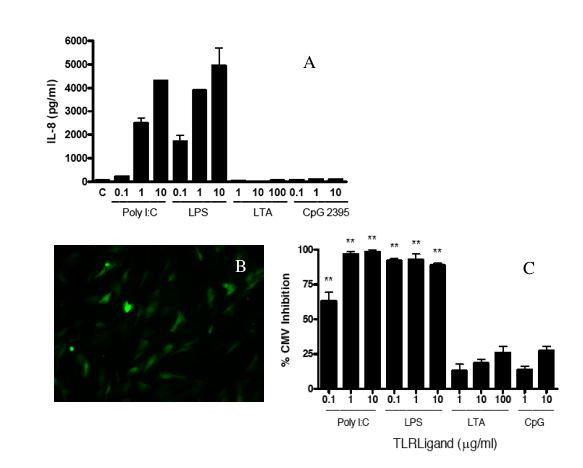
**IL-8 secretion and HCMV inhibition in HFF induced by TLR ligands**. HFF cells were cultured to 95% confluency and stimulated with the indicated doses of TLR ligands or medium control alone (C) for 24 hours. A. Culture supernatants were then collected and assayed for IL-8 by ELISA. IL-8 secretion from one experiment representative of three. Bars represent mean ± SD of triplicate cultures. B and C. After treatment of HFF with medium alone, LTA, Poly I:C, LPS, or CpG 2395 for 24 hours, cells were washed and CMVPT30-gfp was added. After four hours, the virus innoculum was removed and replaced with fresh culture medium. HCMV infection was quantified on day 10 post-infection by counting fluorescent (GFP expressing) cells in each well. B. Shown is a representative culture well from cells treated with medium alone. C. Percent inhibition compared to medium control. Results of one experiment, representative of 3 independent experiments, is shown. Bars represent mean ± SD of triplicate cultures. * indicates P ≤ 0.05 compared to control. ** indicates P ≤ 0.01 compared to control. *** indicates P ≤ 0.001 compared to control.

Initial experiments were performed to determine if ligands for TLR2 (LTA), TLR3 (PolyI:C), TLR4 (LPS), or TLR9 (CpG 2395, a type C oligonucleotide) stimulate human foreskin fibroblasts (HFF) by measuring IL-8 secretion since IL-8 is secreted by a wide variety of cell types in response to stimulation by TLR ligands [21]. HFF secreted IL-8 in response to stimulation with Poly I:C and LPS in a dose dependent fashion (Fig. 1A). In contrast, HFF did not secrete significant levels of IL-8 in response to stimulation with LTA or CpG 2395 (Fig. 1A). Since TLR ligands can induce the secretion of other cytokines in some types of cells, we also assayed HFF supernatants for IL-12 p40, IL-10, TNF-α, and interferon-α. None of these cytokines were detected after stimulation of HFF with LTA, CpG 2395, LPS, or Poly I:C (data not shown).

### TLR3 and TLR4 ligands inhibit HCMV infection in HFF

After stimulation with TLR ligands, HFF were washed and infected with CMVPT30-gfp. After culture, the number of infected cells was determined by quantifying GFP-expressing cells (Fig. 1B). Treatment of HFF with LPS at doses as low as 0.1 μg/ml resulted in a 92% reduction in the number of GFP-positive cells (Fig. 1C). Treatment with 0.1 μg/ml Poly I:C resulted in a 63% reduction in the number of infected cells, while at doses of 1 μg/ml and 10 μg/ml of Poly I:C, >97% reduction in the number of infected cells was observed (Fig. 1C). In contrast, pre-treating HFF with LTA at doses as high as 100 μg/ml or 10 μg/ml CpG did not significantly inhibit infection. Thus, pre-treatment of HFF with TLR3 and TLR4 ligands, but not TLR2 or TLR9 ligands, inhibited HCMV infection.

### Time dependence of TLR stimulation for HCMV inhibition and IL-8 production

**Figure 2:**
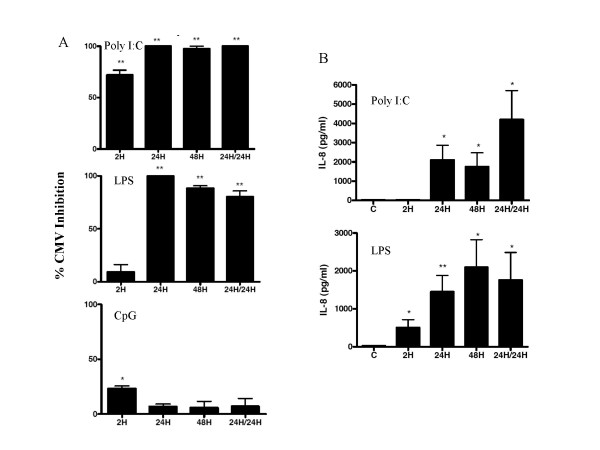
**The effect of time of TLR stimulation on HCMV infection and IL-8 secretion in Foreskin Fibroblasts**. A. Cell monlayers were treated with medium alone, Poly I:C, LPS or CpG 2395 (all at 10 μg/ml) for either 2 hours, 24 hours, 48 hours, or for 24 hours followed by a period of 24 hours with fresh complete medium (24/24 group) and then challenged with CMVPT30-gfp. At day 10 post-infection the number of gfp-expressing foci were determined by fluorescence microscopy and the percent inhibition was calculated based on medium control-treated cells. B. Cells were stimulated with medium alone (control, C) or the indicated dose of the TLR ligands for a period of 2 hours, 24 hours, 48 hours, or for 24 hours followed by a period of 24 hours with fresh complete medium (24/24 group). Culture supernatants were immediately harvested and IL-8 levels determined by ELISA. For both A and B bars represent mean ± SD of triplicate cultures. * indicates P ≤ 0.05 compared to control. ** indicates P ≤ 0.001 compared to control.

The effect of timing of TLR-ligand exposure on inhibition of HCMV infection production was next investigated using the concentration of each TLR-ligand that most effectively inhibited infection in the above experiments. When HFF were exposed to TLR ligands for 2 hours, 72% inhibition of GFP-positive cells was observed in response to Poly I:C, while only 9% inhibition was observed in response to LPS (Fig. 2A). However, both Poly I:C and LPS induced >98% inhibition of HCMV infection when present for 24 hours in cell culture. Anti-HCMV responses induced by Poly I:C and LPS were similar whether cells were exposed to TLR ligands for 24 hours, 48 hours or when stimulated for 24 hours and then incubated in the absence of stimulus for 24 hours before infection (Fig. 2A). When CpG was present for 2 hours, a significant 23% inhibition (p < 0.05) of HCMV replication was noted. However, CpG did not significantly inhibit HCMV when present for 24, 48 hours or 24 hours followed by resting for 24 hours. LTA did not inhibit HCMV at any of the times (not shown). These results show that 24 hours of exposure to Poly I:C and LPS resulted in a maximal anti-HCMV effect.

The effect of time of cell stimulation with TLR ligands on IL-8 production was also determined. Stimulation of HFF cells with Poly I:C for 2 hours did not induce significant secretion of IL-8 above control, although IL-8 was detected after 2 hours exposure to LPS (Fig. 2B). The amount of IL-8 detected after 24 hours of stimulation was higher than after 2 hours for both Poly I:C and LPS (Fig. 2B). The amount of IL-8 detected after stimulation with Poly I:C and LPS for 48 hours was similar to 24 hour stimulation. There was no IL-8 produced by HFF in response to CpG or LTA (data not shown).

### Anti-HCMV effect of TLR3 and TLR4 ligands in HFF is mediated by IFNβ

**Figure 3:**
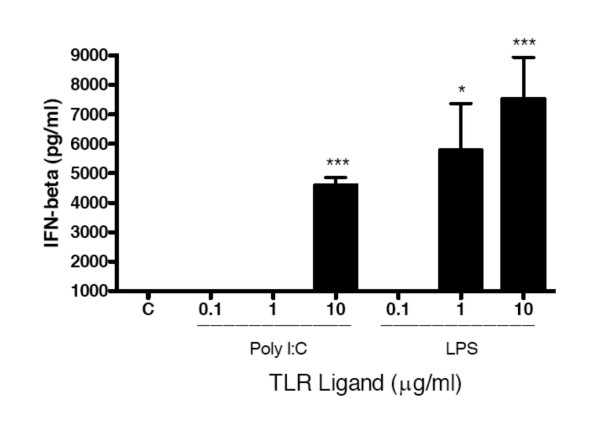
**Poly I:C and LPS induce IFNβ secretion by Foreskin Fibroblasts**. Monolayers of foreskin fibroblasts were stimulated with the indicated doses of TLR ligands or medium alone (Control, C) for 24 hours. Culture supernatants were collected and tested for IFNβ by ELISA. The limit of detection of this assay was 1000 pg/ml. * indicates P ≤ 0.05 compared to control. ** indicates P ≤ 0.01 compared to control. *** indicates P ≤ 0.001 compared to control.

Since fibroblasts are known to produce interferon-beta (IFNβ) in response to stimulation with LPS and Poly I:C [22,23], we hypothesized that the anti-HCMV effects resulting from stimulation of HFF with TLR ligands were mediated by IFNβ. To determine if IFNβ was present, HFF were stimulated with LTA, Poly I:C, LPS, or CpG 2395 for 24 hours and the level of IFNβ was measured in culture supernatants by ELISA. Poly I:C at 10 μg/ml induced detectable IFNβ, while LPS induced detectable levels of IFN-β at 1 μg/ml and 10 μg/ml (Fig. 3). In contrast, LTA and CpG did not induce detectable IFN-β(data not shown).

**Figure 4:**
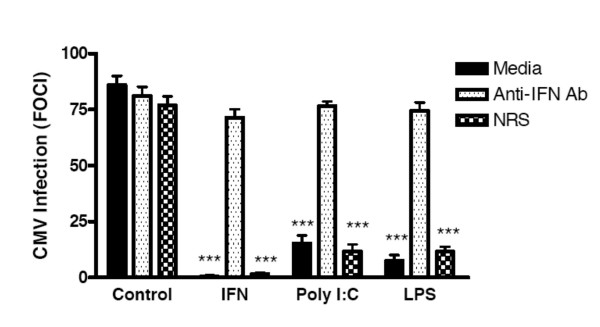
**IFNβ induced by Poly I:C and LPS mediates resistance to HCMV in HFF**. Monolayers of HFF were treated with either medium alone, Poly I:C (10 μg/ml) or LPS (10 μg/ml) for 24 hours. Cells were washed three times and cultured for an additional 24 period in one ml of fresh medium. These conditioned supernatants were collected and incubated in the presence of either medium as a control, rabbit polyclonal anti-IFNβ antibody, or normal rabbit serum for 1 hour at 37°C. Recombinant IFNβ (IFN) was also incubated in the presence of either medium as a control, rabbit polyclonal anti-IFNβ antibody, or normal rabbit serum for 1 hour at 37°C. The treated supernatants were then transferred to wells of confluent HFF fibroblasts and cultured for 24 hours. The conditioned medium was removed and the fresh HFF were challenged with CMVPT30-gfp. Fluorescent cells were then counted on day 10 post-infection (PI). The data shown is representative of 3 independent experiments. *** indicates P ≤ 0.001 compared to control.

We next determined if IFNβ produced by HFF in response to Poly I:C or LPS was responsible for mediating anti-HCMV effects. HFF were stimulated with Poly I:C or LPS for 24 hours, washed, and cultured an additional 24 hours to produce conditioned medium. Conditioned medium was treated with rabbit polyclonal anti-IFNβ antiserum or control antiserum and added to fresh HFF prior to HCMV infection. In the absence of IFNβ neutralizing antibody, Poly I:C-conditioned medium inhibited HCMV replication by 73% and LPS-conditioned medium inhibited HCMV replication by 84% (Fig 4). Addition of anti-IFNβ antibody reduced the ability of Poly I:C and LPS conditioned medium to inhibit HCMV, resulting in only 8% and 20% inhibition, respectively (Figure 4). In contrast, normal rabbit serum did not decrease the inhibition of HCMV infection of Poly I:C- and LPS-conditioned medium (Figure 4). These results show that stimulation with TLR3 and TLR4 ligands induced secretion of IFNβ that inhibited HCMV infection of HFF.

### TLR3, TLR4, and TLR9 ligands induce IL-8 secretion in ectocervical explant tissue

**Figure 5:**
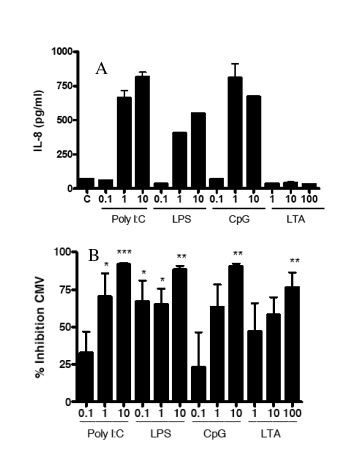
**TLR Ligands induce IL-8 secretion and inhibit HCMV infection in Ectocervical explant tissue**. A. Ectocervial explant tissue was incubated with TLR ligands for 24 hours. Supernatants were removed and assayed for IL-8 by ELISA. The mean ± SD of triplicate cultures is shown from one experiment that is representative of three separate experiments. B. Ectocervical explant tissue was incubated with TLR ligands for 24 hours. Tissues were infected with HCMV and levels of HCMV were assessed by real time PCR after 12 days of culture. Average of three experiments. * indicates P ≤ 0.05 compared to control. ** indicates P ≤ 0.01 compared to control while *** indicates P ≤ 0.001.

The ability of TLR ligands to stimulate cells within ectocervical explant tissue was investigated by measuring IL-8 in culture supernatants. Poly I:C significantly induced IL-8 at 1 and 10 μg/ml (p < 0.001) (Fig 5A). LPS induced detectable IL-8 at all concentrations, although at lower levels than Poly I:C. In contrast to HFF, ectocervical explant tissues secreted IL-8 in response to CpG at 1 and 10 μg/ml. LTA did not induce IL-8.

### TLR2, TLR3, TLR4, and TLR9 ligands inhibit HCMV infection in ectocervical explant tissue

The ability of TLR ligands to inhibit HCMV infection was next evaluated by real-time PCR for HCMV DNA instead of counting fluorescent cells since GFP-positive cells were observed, but difficult to accurately count in the three-dimensional tissue matrix. Ectocervical explant tissue was incubated with Poly I:C, LPS, CpG, or LTA for 24 hours prior to infection and tissues were harvested 12 days after infection to determine HCMV DNA levels. Previous studies indicated that this time point was near the peak of HCMV levels [18]. Both Poly I:C and LPS significantly inhibited HCMV infection at 1 μg/ml and 10 μg/ml (Fig. 5B). However, LPS also inhibited HCMV infection at 0.1 μg/ml (Fig 5). CpG inhibited HCMV infection significantly at 10 μg/ml (p < 0.0001). Surprisingly, LTA inhibited HCMV infection significantly at 100 μg/ml (p < 0.001).

### IFN-β mediates anti-HCMV effect of TLR-ligands in ectocervical explant tissue

**Figure 6:**
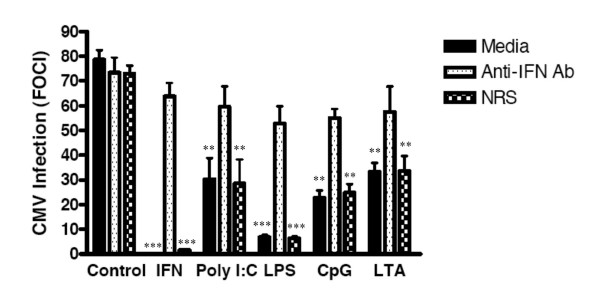
**IFNβ induced in Ectocervical tissue by TLR ligands mediates resistance to HCMV**. Ectocervical tissue was treated for 24 hours with TLR ligands. Tissues were washed three times and cultured for an additional 24 period in one ml of fresh medium. These conditioned supernatants were collected and incubated in the presence of either medium as a control, rabbit polyclonal anti-IFNβ antibody, or normal rabbit serum for 1 hour at 37°C. The treated supernatants were then transferred to wells of confluent HFF and cultured for 24 hours. The conditioned medium was removed and treated HFF were challenged with CMVPT30-gfp. Foci of infection containing gfp-expressing cells were then counted on day 10 post-infection. The data shown is the mean ± SD from one experiment that is representative of 3 independent experiments. ** indicates P ≤ 0.01 compared to control. *** indicates P ≤ 0.001 compared to control.

We next determined whether IFNβ was involved in the anti-HCMV effect of the TLR ligands in ectocervical explant tissue. Conditioned medium was collected after 24 hours of stimulation of ectocervical explant tissue with Poly I:C, LPS, CpG, or LTA. Poly I:C conditioned medium inhibited HCMV infection by 61% and the inhibition was completely reversed by the presence of anti-IFNβ antibody but not control serum (Fig. 6). Although LPS did not induce IL-8 as potently as Poly I:C in ectocervical tissues, LPS conditioned medium inhibited HCMV infection by 91%, and the inhibition was reversed by neutralization of IFNβ (Fig. 6). CpG conditioned medium also significantly inhibited HCMV infection (71%) and inhibition was shown to be dependent on the presence of IFNβ (Fig 6). Although LTA did not induce significant levels of IL-8 in ectocervical tissue, conditioned medium from LTA-treated ectocervical tissues inhibited HCMV infection by 56% and this was reversed by anti-IFNβ. These results demonstrate that IFNβ contributes to the anti-HCMV effect of TLR2, TLR3, TLR4, and TLR9 ligands in ectocervical tissues. No interferon-α was detected in supernatants of TLR-stimulated cultures by ELISA (not shown).

### Expression of TLR by HFF and ectocervical tissue

**Figure 7:**
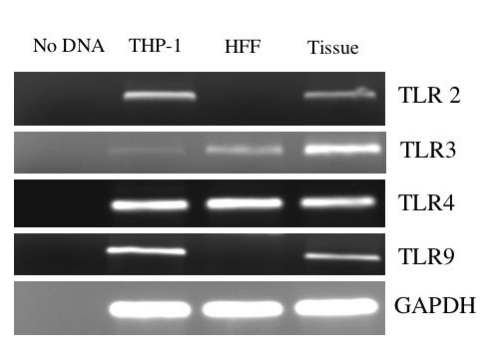
**Expression of TLR by HFF and Ectocervical tissue**. Expression of TLR2, TLR3, TLR4 and TLR9 in cells and tissue was assessed by reverse-transcription PCR. mRNA was isolated from THP-1 monocytic cells, HFF and tissue and reverse transcribed to create cDNA which was subjected to PCR using primers for each of the TLR as well as for GAPDH.

An anti-HCMV response was observed by ectocervical tissue in response to all TLR ligands but in HFF only in response to TLR3 and TLR4 ligands. These findings suggested that ectocervical tissue and HFF differentially expressed TLR. To determine expression of TLR, mRNA from HFF and ectocervical tissue was isolated, reverse transcribed, subjected to PCR and the products visualized on gels. The THP-1 cell line was similarly analyzed since these cells are know to express multiple TLR [24]. Bands were observed after amplification of ectocervical tissue cDNA and THP-1 cells cDNA for all four TLR (Fig. 7). In contrast, for HFF, bands were observed only for TLR3 and TLR4 suggesting a lack of expression of TLR2 and TLR9 by these cells.

## Discussion

Sexually transmitted microbial diseases or bacterial vaginosis expose genital tract cells to TLR ligands. In this study we performed experiments to determine if exposure to defined TLR ligands affects HCMV infection and found that TLR ligands inhibit HCMV infection of both HFF and ectocervical explant tissue through induction of IFNβ. While no previous studies directly investigated the effect of TLR ligand stimulation of cells in vitro on HCMV infection, Sainz et al [25] showed that the pretreatment of HFF with either IFN-α, IFNβ, or IFN-γ inhibited HCMV infection. Several previous studies showed induction of IFNβ in HFF, HEK fibroblasts, and human lung fibroblasts in response to stimulation with Poly I:C [26-28].

While the effect of genital microbial infections on initial HCMV infection of women has not been reported, Ross et al. [4] recently reported that HCMV shedding was found at a higher rate in women with BV than in women with normal flora. Infection with *T. vaginalis*, gonorrhea, and BV were independently associated with intrauterine transmission of HCMV [7]. Thus, these clinical studies show that under some in vivo conditions, HCMV infection can be enhanced by infections with other infectious agents. This suggests that TLR ligands may enhance HCMV infection in vivo since GC, *T. vaginalis *and BV all have TLR ligands (TLR2, TLR4 and TLR2 respectively) associated with their infections [8-10]. The clinical studies contrast with the findings of our in vitro and ex vivo studies where inhibition by defined TLR ligands was observed. A possible explanation for the differences could be that many of the clinical infections are chronic infections that in vitro 24 and 48 hour treatments with TLR ligands fail to accurately model. Also, in vivo adaptive immune responses or other stimuli may be present that affect HCMV that are lacking in vitro. Further studies are needed to understand these apparent differences.

A recent study showed that during infection with murine CMV, virus replicates to higher levels in mice lacking TLR2 [29]. Depletion of Natural Killer (NK) cells eliminated the difference between TLR2-positive and TLR2-negative mice suggesting NK cells were involved in virus suppression in TLR2-positive mice. Also, type 1 interferon was lower in the TLR2 negative mice suggesting a role in virus suppression. The CMV inhibition in mice is different than the in vitro HCMV inhibition described in our study since in the mice no exogenous TLR ligands were given before infection. Intact HCMV virions have been reported to activate TLR2, possibly via glycoproteins B and H [30,31], although murine CMV is not known to have this activity. Iverson et al. [32] showed that human NK cells can suppress HCMV through secretion of IFNβ, and NK cells can be stimulated through certain TLR including TLR2 [33]. In our in vitro studies, no NK cells were present in HFF cultures showing that TLR3- and TLR4-ligands had a direct effect on the HCMV infection targets. However, in ectocervical tissue, it is possible that targets of HCMV infection as well as non-targets, such as immune cells, could have produced interferons. In mice, murine HCMV replicates to higher levels in mice deficient in TLR9 or MyD88 [34,35]. This higher replication is again associated with lower levels of type 1 IFN and decreased NK cell activity. However, mouse embryonic fibroblasts, dendritic cells and macrophges, and human fibroblasts have all been shown to secrete IFNβ in response to stimulation with LPS [22,36-38]. Thus, it is likely that multiple cell types in ectocervical tissues secrete IFNβ and contribute to the anti-HCMV effect.

Another interesting observation made in the current study was that the response pattern to the TLR ligands was different between HFF and ectocervical explants. Anti-HCMV responses in HFF were only found with TLR4 and TLR3 ligands while significant HCMV inhibition was induced by ligands to TLR2, TLR3, TLR4 and TLR9 in ectocervical explants. In our studies, IL-8 was measured to determine the responsiveness of HFF and explants to the TLR ligands. The IL-8 response pattern to the TLR ligands was also different between HFF and ectocervical explants with only TLR3 and TLR4 ligands inducing IL-8 in HFF but TLR3, TLR4 and TLR9 ligands inducing IL-8 in the tissue. Analysis of mRNA indicated that the ectocervical tissue expressed all four of the TLR while HFF only expressed TLR3 and TLR4. Many cell types express restricted repertoires of TLR receptors. For example, many epithelial cells have been observed to lack expression of TLR4 but to respond to TLR2 ligands [39]. This highlights the importance of using models to study HCMV infection that most closely mirror the types of cells that are present in vivo. Cultures of ectocervical tissue have been used to study factors that affect HIV-infection [20] and to assess the interactions of HIV with HCMV [18], but this is the first study to investigate how TLR ligands affect HCMV infection in this tissue.

The inability of a TLR9 ligand to inhibit HCMV in HFF may be due to a lack of expression of TLR9 in these cells. TLR2 is not generally recognized to activate signaling pathways that lead to IFN production and may explain the lack of anti-HCMV effect in HFF due to this TLR ligand [40]. However, TLR2 induced an anti-HCMV effect in ectocervical tissue and this appeared to be dependent on IFNβ. The mechanism for induction of IFNβ by TLR2 in tissues is not known although as mentioned above, some cells may produce IFN in response to TLR2 ligands. Also, stimulation through TLR2 can upregulate a number of molecules involved in anti-viral responses such as TRIF [41] possibly leading to enhanced IFN production by cells due to other stimuli.

In conclusion this study shows that defined TLR ligands inhibit HCMV replication via IFNβ which suggests that different types of flora in the female genital tract can influence HCMV infection. This further suggests that reactivation and shedding of HCMV in the genital tract may be determined by alterations in the normal flora, which results from underlying conditions such as bacterial vaginosis or sexually transmitted diseases.

## Authors' contributions

SH performed all of the cultures experiments and participated in writing of the manuscript. NL obtained and processed cervical tissue and provided direction to the studies. MRZ performed the TLR expression studies. GTS provided overall direction and co-wrote the manuscript. All authors read and approved the final manuscript.
